# Indigenous chicken production in Fiji Islands: knowledge, constraints and opportunities

**DOI:** 10.5713/ab.21.0309

**Published:** 2021-10-29

**Authors:** Titus Jairus Zindove, Archibold Garikayi Bakare, Paul Ade Iji

**Affiliations:** 1 Department of Animal Science, School of Animal and Veterinary Sciences, Fiji National University, P.O Box 7222, Nasinu, Fiji; 2 School of Environmental and Rural Sciences, University of New England, Armidale, New South Wales 2351, Australia

**Keywords:** Constraints, Flock, Indigenous Chickens, Markets, Productivity

## Abstract

**Objective:**

The objective of the study was to understand and document socio-economic characteristics, production parameters, challenges and management practices used by Fijian households which keep indigenous chickens.

**Methods:**

A survey involving 200 households was carried out in coastal and inland communities of Fiji’s wet and semi-dry ecoregions. Data on the influence of ecoregion and location of households relative to the sea on management practices, challenges and productivity of indigenous chickens were analyzed using logistic regression and general linear model of SAS software.

**Results:**

Irrespective of location relative to the sea and ecoregion, households indicated that they kept indigenous chickens for food and income generation. The Welsummer was the most (p>0.05) preferred breed. Households in the semi-dry inland communities had the largest (p<0.05) flocks compared to those in semi-dry coastal communities and the wet region. Chickens in the semi-dry region performed better (p<0.05) than those in the wet region in terms of number of clutches per year and mature live weight. Predators and feed shortages were the biggest challenges faced by households in all areas. The mongoose was ranked as the most (p>0.05) common predator followed by domestic dogs. Most households in the wet ecoregion’s coastal communities housed their chickens at night, whereas communities in semi-dry ecoregion housed their chickens most of the time (p<0.05). In all regions, no households sold their chickens to commercial markets (p>0.05). Households in semi-dry ecoregion were more likely (p>0.05) to sell their chickens at the local market place.

**Conclusion:**

The productivity of local chickens in Fiji is low because of feed shortage, predators such as the mongoose and lack of market linkages.

## INTRODUCTION

In Fiji, poultry production plays a vital role in food security and contributes to over 15% of the agricultural gross domestic product [1]. Poultry products in the island nation are on high demand because they are consumed across cultures, traditions, and religions. Hindus and muslims, who constitute almost half of Fiji’s population, do not eat pork and/or beef and, thus, mainly rely on poultry products [2]. Like most countries in the world, the majority of the poultry in Fiji are chickens [1].

Fiji has an estimated 3.7 million live chickens, which meet approximately 80% of the domestic demand for chicken products [1]. Almost 90% of the chickens are improved breeds (broilers and layers), whereas indigenous breeds constitute less than 10% [1]. This creates a supply deficit for indigenous chickens and their products in Fijian urban and rural areas. In most developing countries, there are trends of consumers in both urban and rural settings preferring products from chickens kept in free-range conditions, which best suits indigenous chicken breeds [3,4]. The same trends could apply in Fiji but there is a need to ascertain the postulations.

Studies in other developing regions such as Africa and Asia, which are characterized by low input agricultural systems just like Fiji, have proved that chickens that are indigenous to the region make substantial contributions to urban and rural household food security as a viable source of meat, eggs and income due to the ease of production [5]. The chickens are adapted to harsh environmental conditions found in most of these countries such as floods, cyclones, extreme temperatures and frequent droughts. In Fiji, the frequency and severity of the impact of phenomena associated with the harsh environmental conditions vary with ecological region and location relative to the sea. There are two main agro-ecological regions, the semi-dry region associated with erratic rainfall and the wet region, which experiences frequent rainfall throughout the year [6,7]. Chicken production in the two regions is likely to differ.

Despite the importance of indigenous chicken and their adaptation to harsh environmental conditions, to our knowledge, no studies have been carried out to characterize, evaluate, understand and develop the indigenous chickens and their production systems in Fiji. The low productivity and market deficit of indigenous chickens in Fiji, as reported by Diarra [2], could be because of lack of comprehensive information on market availability, proper breeding practices and their adaptive characteristics. There is a need to document market opportunities, characteristics, the geographical distribution of indigenous chickens and current production practices by producers. Such information can be the basis for planning the management of indigenous chicken genetic resources at local, national and even regional level. Adoption of intervention programs to improve the production of indigenous chickens depends upon the social, cultural, economic and environmental conditions facing the target population. It is, thus, important to take into consideration constraints faced by local communities when designing and implementing sustainable development programmes that are based on the indigenous chicken resources to benefit resource-poor households. The objective of the current study was, therefore, to document knowledge, constraints and opportunities in the production of indigenous chickens in Fiji.

## MATERIALS AND METHODS

### Study site

The study was carried out in Rakiraki and Nausori districts. Rakiraki is located at 17°22′S and 178°10′ E and is 6 m above sea level. Nausori is located at 18°2′ S and 178°32′ E and is 11 m above sea level [6]. The two districts are located on Fiji’s largest island, Viti Levu. Rakiraki district, located in Ra province, is situated on the leeward side of the island and experiences a tropical semi-dry climate, which is characterized by average annual precipitation between 600 and 1,600 mm, and a dry season that ranges from five to eight months between April and November [6]. In contrast, Nausori district, situated in Tailevu district, experiences a tropical wet climate. The area receives rainfall throughout the year, totalling about 3,500 mm. In both districts, there are only slight variations in daily and seasonal temperatures, with a change of only 2°C to 4°C between the coolest months (July and August) and the warmest months (January to February) [6]. While temperature variations generally tend to be similar in the two agro-ecological regions, they can differ within the agro-ecological regions, with day-time temperatures on the inland areas rising by 1°C to 2°C above the coastal areas [7]. The total amount of rainfall received by inland areas is usually greater than that received by coastal areas within the same agro-ecological region and is more variable in occurrence. Livestock production on natural rangelands is a common agricultural practice in both agro-ecological regions [1].

### Sampling procedure

A stratified random sampling method was used to select households to participate in the survey. Twenty villages were selected from the two districts (10 from Rakiraki and 10 from Nausori) based on their geographical location. In each district, 5 villages were randomly selected in coastal areas and 5 villages in inland areas. A total of 10 households that owned indigenous chickens were then randomly selected from each of the 20 villages. Households with at least four indigenous chickens were considered. Only heads of households or household agricultural decision-makers were allowed to participate in the survey.

### Data collection

Heads of households or household agricultural decision makers were interviewed at their homesteads using a pre-tested structured questionnaire. Only household members aged 18 years or above were selected for the interview. Respondents aged 30 years and below were regarded as young [8]. The interviews were conducted in iTaukei and Fiji Hindi languages by trained enumerators. All interviewees volunteered and consented to participate and approved of the subsequent publication of survey responses. Data collected included demographic information, species of livestock owned, number of mature chickens kept, number of chicks, breeds of indigenous chickens kept, reasons for keeping indigenous chickens, marketing of chicken, feed resource base, performance of the chickens and challenges faced. The study was granted ethical clearance by Fiji National University Committee on Human Research Subjects (CHRS# 6-20).

### Statistical analyses

All data were analyzed using SAS [9]. Chi-square tests were computed to determine the association between production environment and household characteristics with indigenous chicken management practices. The effect of agro-ecological region and location relative to the sea on flock size, breeds kept, challenges to indigenous production, prominent predators, feed resource base, market outlets and performance of the chickens were determined using PROC general linear model of SAS [9]. Binomial logistic regression (PROC LOGISTIC) was used to estimate the probability of a household selling chickens to a specific market outlet and not selling their indigenous chickens to commercial market outlets for a specific reason. The logit model fitted agro-ecological region, location relative to the sea and demographic factors such as age, education level, gender, household size and employment status as predictors.

## RESULTS

### Household characteristics and species owned

Table 1 shows the characteristics of household members interviewed and the livestock species they own. In both wet and semi-dry regions, more than 58% of indigenous chicken owners were male irrespective of location relative to the sea. The majority of the household heads were older than 30 years, employed, had large households and had formal education regardless of geographical location. Households in both coastal and inland communities across the two ecological regions kept cattle, goats, sheep, chickens and ducks. The distribution of indigenous chickens and ducks among inland and coastal communities varied (p<0.05) with agro-ecological region. In the wet region, the flock size of ducks and indigenous chickens was the same (p>0.05) in inland and coastal communities. Inland communities in the semi-dry region had larger (p<0.05) duck and indigenous chicken flocks than their counterparts in wet areas.

### Breed preferences and uses of indigenous chickens

The uses for indigenous chickens did not vary (p>0.05) with agro-ecological region and location relative to the sea (Table 2). Food was ranked as the most important use of indigenous chickens and their eggs across agro-ecological regions and locations relative to the sea. Cash generation was ranked second, followed by gifts and then ceremonies. Respondents in both inland and coastal communities of the semi-dry region ranked cash as the second most important use of eggs from indigenous chickens.

The Welsummer was the most preferred breed irrespective of location (p>0.05; Table 2). The Wyandotte breed was ranked lowly in inland areas, whilst in coastal areas it was ranked highly as a preferred breed (p<0.05; Table 2). Leghorns and Naked-neck breeds were ranked lowly across all locations (p>0.05; Table 2) except in the semi-dry region’s inland communities. The reasons for preferring indigenous chickens varied (p<0.05) with agro-ecological region and location relative to the sea (Table 2). Easy maintenance was ranked as the most important reason for respondents preferring indigenous chickens across all locations (p>0.05; Table 2). Although fighting against predators was ranked relatively high as a reason for the preference of indigenous chickens by coastal communities in the wet region, it was ranked lowly (p<0.05) by their counterparts in the semi-dry areas (Table 2). Good meat quality was ranked second as a reason for preferring indigenous chickens by respondents from all areas except in the wet region’s coastal communities where it was ranked fourth. Tasty eggs and clutch size were ranked lowly by respondents from all areas (p>0.05; Table 2).

### Flock structure and performance of indigenous chickens

The flock size and composition of hens, cocks, growers and chicks in coastal and inland communities found in the two agro-ecological regions are shown in Table 3. Mean flock size ranged from 9 to 14 chickens per household depending on location with an average of 3 cocks. Households in inland communities under the semi-dry agro-ecological region had the largest (p<0.05) flocks and the highest (p<0.05) number of hens, growers and chicks. The average number of hens ranged from 4 to 6 hens per flock.

The growth and reproductive performance of indigenous chickens in the study areas are shown in Table 4. Chickens in the semi-dry area had more (p<0.05) clutches per year than those in the wet region. However, the egg clutch size was the same (p>0.05) across the agro-ecological regions, averaging between 9 and 11 eggs. Likewise, the hatching rate and the number of chicks weaned per clutch did not vary (p<0.05) with agro-ecological region or location relative to the sea. Respondents who lived in inland communities had heavier (p<0.05) mature hens than those living along the coast. In the tropical wet agro-ecological region, respondents in inland communities had heavier (p<0.05) mature cocks. Respondents’ location relative to the sea did not affect (p>0.05) the weight of mature cocks in the semi-dry agro-ecological region.

### Challenges faced and management practices used by indigenous chicken producers

Table 5 presents the rankings of reasons for having small flocks of chickens and constraints faced by indigenous chicken producers in the study areas. The main reason for keeping small flocks was expensive feed, which was ranked highly in both agro-ecological regions (p>0.05). High demand and low growth rate were also ranked highly across all regions (p>0.05). Rank scores for most of the challenges faced by the indigenous chicken producers were the same (p>0.05) across the two agro-ecological regions except for theft, which was ranked high (p<0.05) in inland communities. Predators were ranked as the biggest (p>0.05) challenge followed by feed shortage. Irrespective of location, mongooses were ranked first (p>0.05) as the most common predator with domestic dogs coming second.

Housing and feeding management practices by the indigenous chicken producers are shown in Table 6. In the wet agro-ecological region, the majority of the respondents in coastal communities housed their chickens at night whilst almost half (49%) of the respondents in inland communities housed their chickens most of the time (p<0.05). A larger (p<0.05) percentage of the households in the coastal communities in the wet region did not house their chickens as compared to those in inland communities. In the semi-dry agro-ecological region, more than 40% of the respondents in both coastal and inland communities housed their chickens most of the time (p>0.05). About 20% of the respondents housed their chicken at night irrespective of location (p>0.05). More than 15% of the respondents in both coastal and inland communities did not house their chickens (p>0.05). As shown in Table 6, the respondents used different building materials to construct houses for their chickens. The majority of the respondents in the wet agro-ecological region’s coastal communities used a combination of wood and corrugated iron, whereas in inland communities they mostly used corrugated iron and wire (p<0.05). Less than 6% of the respondents in the wet agro-ecological region used bamboo to construct their chicken houses. More than 80% of the respondents in the semi-dry agro-ecological region’s coastal and inland communities used a combination of wood and corrugated iron to build chicken houses (p>0.05).

The association between feeding practices and location of the households relative to the sea differed (p<0.05) with agro-ecological region. More than 45% of respondents from both coastal and inland communities in the wet agro-ecological region allowed their chickens to scavenge without any supplementary feed (p>0.05). In all regions and locations, most of the respondents used wheat middlingsas a supplement for their chickens (p>0.05).

### Market availability

Overall, 54% of the households were not selling their chickens at all. Nine percent of the selling households sold their chickens at the local market place and to village members whilst 88% sold to village members only. Three percent of the selling households sold their chickens at the main markets and to village members. No households sold their chickens to commercial markets such as hotels, restaurants, supermarkets and schools. The market choices for chickens were influenced by agro-ecological region, respondents’ employment status, and household size (Table 7). Chicken producers in the semi-dry agro-ecological region were 30 times more likely (p<0.05) to sell their chickens at the local market place than to other sources. Households with employed heads were four times more likely (p<0.05) not to sell their chickens whilst the unemployed ones were three times more likely (p<0.05) to sell their chickens to village members. Large households were more likely (p<0.05) to sell their chickens at the local market place.

Twenty-five percent of the households were not selling their chickens to commercial markets because they had a small flock, whilst 20% and 55% of the households did not sell to the commercial markets because they were not aware of the market and never tried, respectively. As shown in Table 8, the reason for not selling chickens to commercial markets was influenced by the agro-ecological region and demographic characteristics. Respondents with small household sizes and those in wet agro-ecological regions were more likely not to sell their chickens to commercial markets because they had small flocks (p<0.05). Respondents in semi-dry agro-ecological region were more likely (p<0.05) to venture into the commercial market. Unemployed and small households were also more likely (p<0.05) not to venture into the commercial market.

## DISCUSSION

Understanding the current production status and constraints faced by indigenous chicken producers is important in the designing and implementation of indigenous chicken-based development programmes, which can improve the resilience of vulnerable households. The finding that the majority of the indigenous chicken owners are males and older than 30 years is in contrast to the common trend in communal livestock production where women and youths mostly own and manage poultry, whilst large stocks such as cattle are largely owned by men [10]. The finding herein could be an indication that, in Fiji, women and youths, still face obstacles that prevent them from owning at least chickens among the livestock species. There is, therefore, a need to intensify the integration of gender and age issues into livestock production programmes.

The findings that the flock size of chickens, sheep and goats, and herd size for their cattle did not vary with agro-ecological region and location relative to the sea was not envisaged. Martin et al [11] reported that rural households in the coastal areas focus more on fishing activities than livestock production. Thus, their livestock flocks and herds are expected to be small. Wet regions have high availability of livestock feed resources [12], hence a positive correlation between rainfall and livestock numbers was expected. The sea also affects agricultural activities of coastal communities because of salinity. In dry areas, dry spells and droughts drive soil salinization on the coastline, resulting in a shortage of livestock feed resources, especially for scavenging livestock such as chickens and ducks [13]. This could explain the finding herein that coastal communities in dry areas had smaller duck and indigenous chickens flock sizes.

The uses of indigenous chickens in this study are similar to the findings by Tadele et al [14] and Moussa et al [15] who reported that, in the communal areas of developing regions, indigenous chickens and their eggs are mainly used as food and cash-generating assets. It is important to encourage indigenous chicken producers to prioritise the hatching of eggs if they are to improve their flocks. Contrary to previous studies across several sites in the tropics where the Naked-neck and the Leghorn were among the preferred breeds [16], our study shows that the Welsummer is the most preferred breed in Fiji. Although there is limited, if any, empirical evidence on the maintenance requirements and the meat quality of the Welsummer chicken breed, the most important reason for preferring the common breeds was easy maintenance followed by meat quality. The high rankings in preference for Leghorns and Naked-neck breeds in semi-dry inland communities might be an adaptation strategy as their hardiness matches the harsh environmental conditions in the area. The Leghorn and Naked-neck breeds are well-known for their adaption to extreme temperatures and excellent scavenging capabilities [10]. The adaptive, productive and meat quality traits of the Welsummer breed under Fiji’s communal production systems warrants investigation.

In agreement with findings herein, various studies conducted on indigenous chicken production in communal areas showed flock sizes ranging from 10 to 20 [10,17,18]. The observed large number of cocks relative to the flock sizes can be ascribed to the fact that households always keep many cocks in case they might lose some of the cocks to predation or theft [18]. The smaller number of hens in the flocks agrees with previous reports by Mtileni et al [17] that households’ flocks are mainly composed of growers. It can also be attributed to the finding herein that households mainly keep their chickens for cash generation, so they sell mature chickens and retain the growers. Long-term studies are required to ascertain the flock dynamics and strategies to improve the composition of hens in the households’ flocks.

The finding that the flock size varied with location agrees with Mtileni et al [17] who highlighted that the flock sizes of chickens under the scavenging system is highly variable, depending on resource availability and management practices. Despite ranking hatching highly as a use of eggs, households in the wet regions had smaller flock sizes than those in the inland communities of tropical semi-dry regions. This could be an indication of high mortality rates, predation, theft, low annual egg production or low hatchability. Similarly, Mwalusanya et al [19] found out that flock sizes are large in warm dry zones. Tropical dry zones are characterized by plenty of scavenging land for the chickens and limited crop production activities compared to tropical wet areas [12], so the households can focus on managing their chickens. The same reasons could explain the observation that chickens in tropical semi-dry areas had more laying cycles than those in tropical wet areas. Similar reasons can also explain why coastal communities in the semi-dry agro-ecological region had small flocks. Households in coastal regions prioritize fishing activities to livestock production [11]. Soil salinization on the coastline also results in the shortage of crop-based livestock feed resources [13] for the scavenging chickens. This could explain why inland communities had heavier mature chickens. The reasons behind the possible adverse effects of the sea on indigenous chicken production need to be ascertained.

High feed costs were highlighted as the major reason why households had small flocks. This is in agreement with reports by Mtileni et al [17] who highlighted that low smallholder chicken productivity is due to the fact that the resource-poor households cannot afford to buy supplementary feed. Therefore, Fiji’s chicken feed resource base needs to be broadened, especially in wet regions and coastal communities. Utilisation of indigenous chicken breeds with low maintenance feed requirements is also advisable. High demand and low growth rate were also highlighted as other important reasons why the flocks were small. Although there is no published data in Fiji, studies in other developing countries have shown that the demand for indigenous chickens exceeds supply [20]. Kamau et al [20] indicated that, despite their adaptability, indigenous chicken breeds are usually constrained by slow growth and maturity rate resulting in small flocks under free-range systems. Improvement of the growth performance of the indigenous chickens, therefore, remains crucial. Although feed shortage was highlighted as the major reason for households having small flocks, predation was ranked as the biggest challenge. In most studies, predation is ranked highly as a challenge to chicken production under scavenging systems but it usually comes after feed shortage, and diseases and parasites [17,18,20]. Unexpectedly, diseases and parasites were not mentioned as challenges to indigenous chickens in Fiji. Fiji experiences a hot and wet climate which is suitable for disease transmission and parasites. Therefore, empirical data on the prevalence of diseases and parasites for indigenous chickens in Fiji are required.

The result that the mongoose was the most common predator agrees with the findings of Patbandha et al [21] who reported the mongoose as the most important factor in the death of chickens in India. Although there are no similar studies on chickens in Fiji and nearby Island countries, the mongoose has been reported to be a huge challenge as an invasive species in Fiji [22]. It was unexpected that domestic dogs are considered problematic predators for indigenous chickens in Fiji, as they are not usually referred to as predators under scavenging systems [17,21]. Domestic dogs usually do not kill chickens because they are bred and trained to protect livestock [23]. However, if the domestic dogs are not trained and/or fed properly they can be a common cause of mortality because they have easy access to the chickens [23]. This could also be due to the large number of stray dogs in Fiji. There is need to come up with proper management strategies for the domestic dog population in Fiji to reduce their threat to livestock including scavenging chickens and, thus, food security.

The finding in this study that wheat middlingsis the main feed source for the chickens contradicts descriptions of the scavenging systems in literature. Scavenging is the main source of indigenous chickens feed in most communal areas with occasional supplementation with kitchen leftovers and cereal grains during harvest time [14]. Chickens under free-range systems mostly scavenge for feed picking on feed sources such as crops, locusts, insects and worms [24]. Roots and tuber crops were mentioned as common resources for the chickens in Fiji’s tropical semi-dry areas. This observation concurs with reports by Abegaz and Gemechu [25] who reported that, in tropical regions, root and tuber crops are common feed sources for scavenging chickens. The fact that the households did not mention scavenging feed resource base components such as worms and locusts indicate that they might not be aware of some of the things their chickens feed on during scavenging. The same reason might be the explanation to the unexpected result that insects and coconuts were lowly ranked as feed sources for chickens. Coconut residues have been reported to be a common feed source of scavenging chickens in tropical climates [26]. There is a need to characterize and/or identify the feed resources scavanged by indigenous chickens in Fiji in order to determine the nutritional limitations and, thus, make supplementation decisions.

The observed variation of chicken housing systems with location of the households was not envisaged. Mtileni et al [17] and Okeno et al [18] reported that, although housing material might differ, housing system for communal indigenous chicken producers is relatively similar across regions. Contradicting results could be because of differences in the livelihoods of the respondents. Coastal communities focus more on fishing activities than livestock production [11] and, thus, spend less time managing their livestock. Fishing, coupled with crop farming, consumes most of the time of households in coastal areas of tropical wet regions [11]. This could explain why coastal communities in the wet regions housed their chickens at night only. Integrating fish and scavenging chickens can be suggested as an alternative to increase indigenous chicken production in these areas. The dominance of the housing systems where chickens are housed most of the time observed in this study contradicts literature, which specifies that, under scavenging systems, chickens are left to search for feed for the whole day [17,18,25]. Prolonged housing, coupled with feed shortage, could be among the major reasons of low productivity of indigenous chickens in Fiji. The finding that a small portion of the indigenous poultry owning households did not provide any housing for their chickens tallies with findings by Badubi et al [27] who reported that, in areas without predators that can climb trees, chickens roost on the treetops or any raised items at night. This, however, can be a challenge in Fiji considering that the mongoose, which is the main predator, can climb trees and any other elevated areas [22]. Extension services on housing practices for indigenous chicken producers are necessary.

The use of wood and corrugated iron for housing chickens in Fiji is comparable to reports by Moussa et al [15] that scavenging chicken houses are usually made of materials that are easily available in the area such as scrap and wood. A good housing structure should, however, protect the chickens from harsh weather and predators [17]. Considering that Fiji is prone to cyclones and floods, wood and corrugated iron might not be the best material for constructing chicken houses since they can be easily blown away by the wind. Brick housing structures have been reported to be the most effective in protecting chickens from predators and harsh weather conditions [17].

The majority of the households indicated that their chickens were not for sale despite cash generation being highlighted as one of the main reasons for keeping the chickens. This leads to the suggestion that the chickens are only sold when there is need for cash. These findings agree with Okeno et al [18] who concluded that, under communal production systems, indigenous chickens are only sold when there is a need for money by the households. The selling of chickens by communal households is associated with socio-economic factors such as availability of labour and income [17]. The observation that almost all households who sold their chickens sold to village members validates earlier assertions of this practice by Hailemichael et al [28] who linked it to the small size of flocks. Besides small flocks, the households are not aware of the commercial outlets. Lubandi et al [29] reiterated that, in communal areas, indigenous chickens are produced without a clear market plan of time to buyer and sale price. Therefore, there is a need to assist the households in scaling up the indigenous chicken production and adopting a business model targeting commercial markets such as hotels, restaurants, supermarkets, and schools.

## CONCLUSION

In Fiji, the Welsummer is the most preferred indigenous chicken breed because it is easy to maintain. Indigenous chickens are mostly reared under scavenging systems and are mainly kept for household consumption and sale when the need arises. From the study, it is clear that the agro-ecological region and location of the household relative to the sea affect management practices and productivity of the indigenous chickens. Households in the semi-dry region’s inland areas have the largest flocks. Chickens in the tropical semi-dry agro-ecological region and inland communities perform better in terms of clutch frequency and growth rate. The low productivity of indigenous chickens is associated with constraints such as predators and feed shortage, which needs urgent management intervention strategies. There is a need for control strategies for mongooses and stray dogs, which are the most common predators. There is a need to sensitize farmers to construct chicken houses with cheap but strong materials such as bricks and bamboo. Households in Fiji mostly rely on wheat middlingsto supplement their indigenous chickens which is expensive. Households in tropical wet ecological regions and in coastal areas do not give their chickens enough time to scavenge; they house them for longer periods. The households are not selling their chickens to commercial markets and, thus, there is a need to improve productivity and create market linkages.

## Figures and Tables

**Table 1:** Characteristics of the respondents and mean herd/flock sizes (±standard deviation) of livestock species kept

Class	Wet region		Semi-dry region	
	
	Coastal	Inland	Costal	Inland
Gender (%)				
Males	79.17	79.22	75.71	58.62
Females	20.83	20.78	24.29	41.38
Household head age (%)				
Young (<30 years)	4.17	7.79	7.27	10.53
Old (>30 years)	95.83	92.21	92.73	89.47
Level of education (%)				
No formal education	21.74	31.58	29.09	21.05
Formal education	78.26	68.42	70.91	78.95
Household size (%)				
Large (>5 members)	54.17	63.16	55.36	68.42
Small (<5 members)	45.83	36.84	44.64	31.58
Occupation (%)				
Unemployed	52.17	65.75	75.47	84.21
Employed	47.83	34.25	24.53	15.59
Herd/flock size				
Cattle	2.0±6.00	6.3±1.41	5.4±1.00	7.8±1.39
Goats	3.7±5.82	9.9±2.06	12.9±3.76	13.2±7.13
Sheep	3.0±2.54^b^	6.7±1.80^ab^	4.3±0.92^b^	8.0±1.13^a^
Chickens	15.8±2.71	16.4±1.43	15.6±1.74	15.6±2.74
Indigenous chickens	10.1±1.72^b^	10.8±0.89^b^	8.8±1.36^b^	13.6±1.56^a^
Ducks	14.1±12.05^ab^	12.0±2.38^b^	9.7±4.06^b^	22.5±6.42^a^

a,b Values with different superscripts, within a row, are statistically different (p<0.05).

**Table 2:** Mean rank scores±standard error (ranks)^1)^ for uses of indigenous chickens, chicken breeds owned and reasons for preferring indigenous breeds

Class	Wet region		Semi-dry region	
	
	Coastal	Inland	Costal	Inland
Uses of indigenous chickens				
Food	1.1±0.09 (1)	1.2±0.05 (1)	1.2±0.06 (1)	1.2±0.09 (1)
Cash	1.6±0.13 (2)	1.7±0.07 (3)	1.8±0.07 (2)	1.7±0.12 (2)
Gifts	2.3±0.46 (3)	2.6±0.25 (2)	3.0±0.01 (3)	3.0±0.01 (3)
Ceremonies	2.7±0.30 (4)	2.8±0.11 (4)	3.1±0.26 (4)	4.0±0.69 (4)
Uses of eggs from indigenous chickens				
Food	1.2±0.08 (1)	1.1±0.04 (1)	1.1±0.05 (1)	1.2±0.08 (1)
Cash	1.6±0.14 (2)	1.8±0.09 (3)	1.8±0.07 (2)	1.7±0.12 (2)
Gifts	2.5±0.50 (4)	2.5±0.50 (4)	3.0±0.01 (4)	3.0±0.01 (3)
Hatching chicks	2.0±0.33 (3)	1.6±0.27 (2)	2.6±0.33 (3)	4.0±0.58 (4)
Indigenous chicken breeds owned				
Naked-neck	1.4±0.24^b^ (4)	1.3±0.10^b^ (3)	1.9±0.24^a^ (4)	1.5±0.17^ab^ (2)
Welsummer	1.2±0.20 (1)	1.0±0.10 (1)	1.3±0.10 (1)	1.0 ± 0.38 (1)
Sussex	1.3±0.39^bc^ (3)	1.1±0.14^c^ (2)	1.8±0.31^ab^ (3)	2.2±0.48^a^ (4)
Wyandotte	1.3±0.36^b^ (2)	2.1±0.16^a^ (4)	1.7±0.19^b^ (2)	2.3±0.34^a^ (5)
Leghorns	1.6±0.41 (5)	2.2±0.23 (5)	2.0±0.39 (5)	1.7±0.71 (3)
Reasons for preferring indigenous chickens				
Good meat quality	1.5±0.15 (4)	1.3±0.08 (2)	1.4±0.10 (2)	1.5±0.17 (2)
Tasty eggs	2.5±0.33 (6)	2.3±0.13 (3)	2.2±0.10 (3)	2.4±0.15 (4)
Large clutch size	2.0±0.82 (5)	3.0±0.41 (6)	3.5±0.81 (6)	3.2±0.16 (6)
Good mothering ability	1.1±0.64^a^ (2)	2.9±0.21^b^ (5)	2.8±0.14^b^ (4)	2.7±0.22^b^ (5)
Easy maintenance	1.0±0.25 (1)	1.0±0.22 (1)	1.2±0.12 (1)	1.4±0.21 (1)
Fight against predators	1.3±0.73^b^ (3)	2.6±0.33^a^ (4)	3.1±0.36^a^ (5)	2.0±0.80^ab^ (3)

1) The lower the rank of a use, reason or breed, the greater is its importance.

a–c Values with different superscripts, within a row, are statistically different (p<0.05).

**Table 3:** Distribution of the flock size and structure in indigenous chickens in Fiji’s coastal and inland areas

Parameter		Wet region	Semi-dry region
Flock size^1)^	Coastal	10.1±1.72^b^	8.8±1.36^b^
	Inland	10.8±0.89^b^	13.6±1.56^a^
Hens	Coastal	4.9±0.91^ab^	4.5±0.50^b^
	Inland	4.4±0.51^b^	5.9±0.87^a^
Cocks	Coastal	3.3±0.54	2.8±0.33
	Inland	2.8±0.30	3.4±0.57
Growers and chicks	Coastal	8.1±2.47^b^	6.2±1.64^b^
	Inland	9.2±1.38^b^	14.8±2.74^a^

1) Mean flock size excluded chicks.

a,b Values of the same parameter with different superscripts are statistically different (p<0.05).

**Table 4:** Growth and reproductive performance of indigenous chickens in Fiji

Parameter		Wet region	Semi-dry region
Clutches/yr/hen	Costal	2.8±0.31^b^	3.5±0.15^a^
	Inland	3.2±0.16^ab^	3.6±0.26^a^
Eggs/clutch	Coastal	9.5±0.80	10.7±0.42
	Inland	10.5±0.45	9.3±0.75
Chicks hatched/clutch	Coastal	8.2±1.10	6.6±0.51
	Inland	7.1±0.78	6.0±0.76
Chicks weaned/clutch	Costal	3.8±1.08	5.1±0.43
	Inland	3.8±0.67	5.0±0.68
Body weight of mature hens (kg)	Coastal	1.5±0.08^a^	1.4±0.07^a^
	Inland	1.7±0.04^b^	1.7±0.12^b^
Body weight of mature cocks (kg)	Coastal	1.5±0.08^a^	2.3±0.07^c^
	Inland	1.7±0.04^b^	2.3±0.13^c^

a–c Values of the same parameter with different superscripts are statistically different (p<0.05).

**Table 5:** Mean rank scores±standard error (ranks) for reasons for having small flocks and challenges faced by indigenous chickens producers in Fiji

Class	Wet region		Semi-dry region	
	
	Coastal	Inland	Costal	Inland
Reasons for having small flock				
High demand	1.4±0.22 (1)	1.5±0.16 (2)	2.2±0.26 (3)	2.0±1.24 (3)
No space	3.0±0.72 (4)	1.9±0.55 (4)	3.1±0.51 (7)	4.0±1.54 (6)
Prefers small flock	3.0±1.22 (4)	2.5±0.50 (7)	3.0±0.02^a^ (5)	4.0±0.08^b^ (6)
Low hatching rate	4.0 ± 0.98 (7)	2.1±0.40 (6)	2.7±0.58 (4)	2.5±0.56 (4)
Low growth rate	2.3±0.38 (3)	1.6±0.20 (3)	2.0±0.20 (2)	1.0±0.50 (1)
High mortality	3.0±0.67 (4)	2.0±0.30 (5)	3.0±0.55 (5)	3.0±1.66 (5)
Feed is expensive	1.5±0.33 (2)	1.3±0.22 (1)	1.5±0.12 (1)	1.2±0.16 (2)
Challenges faced				
Feed shortage	1.6±0.15 (2)	1.6±0.11 (2)	1.6±0.12 (2)	2.0±0.41 (2)
Lack of market	3.4±0.26 (4)	3.3±0.25 (6)	2.7±0.30 (3)	-
No housing	3.7±0.36 (5)	3.0±0.30 (4)	2.7±0.37 (4)	-
Cyclones	3.8±0.41 (6)	3.4±0.29 (5)	3.1±0.23 (5)	3.0±0.79 (4)
Theft	3.4±0.23^a^ (3)	2.3±0.21^b^ (3)	3.7±0.36^a^ (6)	2.1±0.35^b^ (3)
Predators	1.2±0.09 (1)	1.1±0.05 (1)	1.2±0.07 (1)	1.1±0.12 (1)
Common predators				
Domestic dogs	1.8±0.16 (2)	1.9±0.11 (2)	1.8±0.13 (2)	1.7±0.25 (2)
Feral dogs	3.0±0.38 (3)	2.6±0.27 (4)	3.3±0.30 (4)	2.8±0.35 (4)
Birds	-	2.0±0.11 (3)	2.2±0.26 (3)	1.8±0.20 (3)
Mongoose	1.1±0.08 (1)	1.1±0.04 (1)	1.2±0.07^a^ (1)	1.7±0.12^b^ (1)

a,b Values with different superscripts, within a row, are statistically different (p<0.05).

**Table 6:** Frequencies (%) of indigenous chicken producers in Fiji using different housing and feeding management practices

Variable	Wet region			Semi-dry region		
	
	Coastal	Inland	Chi-square	Coastal	Inland	Chi-square
Housing			0.003			0.48
No housing	29.17	12.99		16.07	31.58	
Housing at night	54.17	35.06		19.64	21.05	
Housed most of the time	8.33	49.35		55.36	42.11	
Permanent housing	8.33	2.60		8.93	5.26	
Housing construction material			0.04			0.14
Wood and corrugated iron	62.50	36.36		3.57	15.79	
Corrugated iron and wire	20.83	51.95		92.86	84.21	
Mud and corrugated iron	12.50	6.49		-	-	
Bamboo	4.17	5.19		3.57	-	
Feeding system			0.46			0.04
Scavenging only	58.33	46.75		56.36	84.21	
Feeding whilst housed	8.33	9.09		1.82	5.26	
Supplementary feeding	33.33	46.75		41.82	10.53	
Type of supplement			0.11			0.54
Wheat middlings	58.33	88.31		83.84	89.47	
Homemade ration	25.00	5.19		3.64	0.00	

**Table 7:** Odds ratio estimates, lower and upper confidence interval of households selling indigenous chickens to different market outlets

Predictor	Not selling			Local market place			Village members		
		
	Odds	LCI	UCI	Odds	LCI	UCI	Odds	LCI	UCI
Agro-ecological region (wet vs semi-dry)	0.6^ns^	0.23	1.37	0.03^*^	2.28	43.44	0.9^ns^	0.40	1.89
Location (coastal vs inland)	0.9^ns^	0.40	2.16	3.0^ns^	0.52	16.96	0.8^ns^	0.36	1.60
Age of household head (old vs young)	0.7^ns^	0.20	2.51	0.3^ns^	0.03	3.26	1.5^ns^	0.46	4.86
Employment status (employed vs unemployed)	3.8^*^	1.56	9.24	0.2^ns^	0.01	2.86	0.3^*^	0.15	0.83
Gender of household head (male vs female)	1.2^ns^	0.51	3.06	1.6^ns^	0.18	14.26	0.8^ns^	0.39	1.86
Household size (large vs small)	0.8^ns^	0.33	1.75	11.0^*^	1.76	68.38	1.4^ns^	0.67	2.93
Level of education (formal education vs no formal education)	2.1^ns^	0.86	5.08	0.4^ns^	0.08	1.89	1.0^ns^	0.47	1.98

LCI, lower confidence interval; UCI, upper confidence interval.

Higher odds ratio estimates indicate greater difference in likelihood of a household selling the indigenous chickens to a specific market place.

* p<0.05;

ns p>0.05.

**Table 8:** Odds ratio estimates, lower and upper confidence interval of reasons for not selling indigenous chickens to commercial markets such as supermarkets, hotels and restaurants

Predictor	Small flock size			Not aware of market			Never tried		
		
	Odds	LCI	UCI	Odds	LCI	UCI	Odds	LCI	UCI
Agro-ecological region (wet vs semi-dry)	2.9^*^	1.17	7.21	0.5^ns^	0.18	1.28	0.4^*^	0.17	0.98
Location (coastal vs inland)	3.1^ns^	0.32	7.47	1.0^ns^	0.40	2.54	0.5^ns^	0.22	1.13
Age of household head (old vs young)	0.9^ns^	0.25	3.18	4.5^ns^	0.52	39.12	1.2ns	0.36	4.19
Employment status (employed vs unemployed)	1.1^ns^	0.42	2.92	0.8^ns^	0.26	2.42	0.9^*^	0.35	2.29
Gender of household head (male vs female)	0.7^ns^	0.32	1.69	0.5^ns^	0.20	1.20	0.9^ns^	0.40	2.08
Household size (large vs small)	0.4^*^	0.16	0.92	0.6^*^	0.22	1.41	3.2^*^	1.42	7.43
Level of education (formal education vs no formal education)	1.1^ns^	0.51	2.78	1.4^ns^	0.55	3.68	1.1^ns^	0.50	2.31

LCI, lower confidence interval; UCI, upper confidence interval.

Higher odds ratio estimates indicate greater difference in likelihood of a household selling the indigenous chickens to a specific market place.

* p<0.05;

ns p>0.05.
